# Return to work in miners following anterior cruciate ligament reconstruction

**DOI:** 10.11604/pamj.2015.22.173.7979

**Published:** 2015-10-22

**Authors:** Ugur Tiftikci, Sancar Serbest, Cem Yalin Kilinc, Gül Öznur Karabicak, Özge Vergili

**Affiliations:** 1 Kirikkale University, Faculty of Medicine, Department of Orthopaedics and Traumatology, Kirikkale, Turkey; 2 Mugla University, Faculty of Medicine, Department of Orthopaedics and Traumatology, Mugla, Turkey; 3 Hacettepe University, Faculty of Health Sciences, Department of Physical Therapy and Rehabilitation, Ankara, Turkey; 4 Kirikkale University, Faculty of Health Sciences, Department of Physical Therapy and Rehabilitation, Kirikkale, Turkey

**Keywords:** Anterior cruciate ligament, injury, knee, reconstruction, miner, return to work

## Abstract

**Introduction:**

The aim of the study is retrospectively investigated durations for returning to work following anatomic ACL reconstruction by hamstring autograft in miners and the reasons in patients who were delayed to return to work.

**Methods:**

Miners with symptomatic anterior cruciate ligament rupture underwent arthroscopic reconstruction. Patients were evaluated in terms of range of motion (ROM) values; Lysholm, Cincinati and Tegner activity scales; laxity testing and complications. By modifying the method used by Fitzgerald et al. we decided for the criteria returning to work.

**Results:**

Thirty three patients were evaluated with mean followup of 22.7 ± 8.3 months (range 13-46 months). Mean age at the surgery was 27.8 (18-38) years. Lysholm, Cincinati and Tegner activity scales were signifi cantly higher from preoperative scores (Lysholm scores: preoperative: 60.7 ± 12.5, postoperative: 90.3 ± 4.8 (P < 0.001); Tegner activity scores: Preoperative 3.5 ± 1.4, postoperative: 6.2 ± 1.5 (P < 0.001); Cincinati scores: Preoperative: 14.8 ± 5.3, postoperative: 26.9 ± 1.6 (P < 0.001). The average time for returning to work was determined as 15,3 ± 4 weeks. There was no significant difference for knee scores and time for returning to work between patients with meniscal injuries and don't have meniscus lesions.

**Conclusion:**

The reasons for delays in returning to work was work accident. Hematoma or effusion and pain inside the knee were the most significant reason which affected returning to work.

## Introduction

Anterior cruciate ligament (ACL) injury is the most commonly injury among ligament injuries of the knee region. ACL reconstruction surgery is the most frequently performed operation by the orthopedic surgeons [1]. This injury has been shown to commonly cause joint effusion, muscle weakness, loss of joint function, joint instability, pain and osteoarthritis at young ages, between 10-20 years of age [2–4]. The management of ACL injuries is both conservative and surgical. However, the up-to-date management has increased the tendency to surgical treatment in people dealing with sports actively, young people, active people and heavy-workers [1, 2, 5]. The most preferred and up-to-date method in surgical treatment is "medial portal anatomic ACL reconstruction by hamstring autograft" [5]. In this study, we retrospectively investigated durations for returning to work following anatomic ACL reconstruction by hamstring autograft in miners and the reasons in patients who were delayed to return to work.

## Methods

33 of 38 miners who had undergone arthroscopic ACL reconstruction by using anatomic medial portal between 2009 and 2014 were included in the study, through their hospital records. Patients with multiple ligament injuries, total meniscectomy, contralateral knee or lower extremity injury, who were aged over 40 years, and whose records were inaccessible were excluded from the study. Laxity tests (Anterior drawer, Lachmann, Pivot shift), effusion and tenderness were assessed. ROM value was scored using goniometer and differences in maximum ROM between involved and contralateral sides were measured. Lysholm, Cincinati and Tegner activity scale was scored after 6 months of the surgery. However, since KT-1000 device was absent, arthrometer test was not performed. The time for starting to work and the reasons for delay in returning to work were identified through hospital records. By modifying the method used by Fitzgerald et al. [6], we decided for the criteria returning to work by knee function test, single-legged hop test score, Lachman test, anterior drawer test and pivot-shift test in miners. This study was approved by the institutional ethical committee; all patients were informed and the study was performed in accordance with the declaration of Helsinki as revised in 2000.

### Surgical Technique

1 gr of first-generation cephalosporin was administered to all patients half an hour before the operation for prophylaxis. This prophylactic antibiotic was continued for 24 hours, until the drain was removed. For confirmation of the ACL tear, in all patients, before surgery, the arthroscopic assessment of the knee was performed in the supine position under general or regional anesthesia and under pneumatic tourniquet. The associated pathologies were identified and necessary treatments were made arthroscopically. By using a 4 cm longitudinal incision, at the medial of tibial tubercle and over pes anserinus, gracilis and semitendinosus tendons were reached. By using a tendon stripper, tendons were excised and the attached muscle tissues were cleaned. By folding the tendons four times, the diameter of the graft was determined. From the appropriate medial portal for this diameter, anatomic femoral tunnel and then the tibial tunnel were prepared; then, by the help of guidewires, graft was positioned into the tunnels. At the proximal side, the graft was fixed to the femur by endobutton system (ToggleLoc ZipLoop, Biomet Sport Medicine). Distally, while the knee was positioned in 10°-20° flexion and graft was being held tightly, the fixation of the graft inside the tunnel was accomplished by one BioComposite Interference Screw (Biomet Sports Medicine) and one staple U nail.

### Postoperative management protocol

Isometric quadriceps strengthening and flexion exercises were immediately started in all patients. Bearing weight as much as they can tolerate, the patients were permitted to walk using knee braces and crutches. At the 2-14 days our goals were obtained full extension, minimize swelling, allow wound healing, maintain active quadriceps control and achieve 90° of flexion. At the 2-6 weeks, we aimed to increase flexion to 135 degrees and increase muscle tone. Daily activities were allowed to be gradually increased until the 4th week. In all patients, compelling early rehabilitation was started [7, 8]. In cases of muscular atrophy and functional disability of the knee, advanced physical rehabilitation therapy was used. Sports and active exercises were permitted after postoperative 2 ½ months.

### Statistical analysis

Statistical analysis was carried out by using the Statistical Package for the Social Sciences (SPSS) software version 15.0 (SPSS Inc., Chicago, USA) and data are presented as mean ± standard deviation comparisons paired t-test, unpaired t-test and Mann-Whitney U-test was performed. P < 0.05 was considered to be significant.

## Results

The average age of patients was 27.8 (18-38). All patients were male. The reasons for ACL injury was work-accident in 16, sports injury and other reasons in 17 patients. The ACL tear was at the right knee in 20 patients and left knee in 13 patients. In the postoperative period Lysholm, Cincinati and Tegner activity scores were significantly higher from preoperative knee scores (Table 1). In 12 patients, medial, in 7 patients lateral, and in 6 patients both medial and lateral meniscal tears were present. In 3 patients having meniscal injuries, partial meniscectomy was performed. In 16 patients, meniscus was sutured using all in site technique (Sequent, Linvatec. In 3 patients, medial parapatellar plica excision was performed. The mean ROM deficit (involved vs. contra knee) was significant difference for knee scores, ROM deficits and time for returning to work of the patients with work-accident and sports injury (Table 2). The average time for returning to work was determined as 15,3 ± 4 (range 10-27) weeks. The reasons for delays in returning to work was work accident. Hematoma or effusion, pain inside the knee were the most significant reason which affected returning to work (Table 2). During the last follow-up, the difference of muscular atrophy was found as 4.2 cm in average. There was no significant difference for knee scores and time for returning to work between patients with meniscal injuries and don't have meniscus lesions (Table 3). In 4 patients, for developing intra-articular effusion during the procedure, effusion drainage was performed. In one patient, at postoperative 1st month, stable U nail was removed because of superficial infection.

**Table 1:** Knee scores

Knee scores	Preoperative	Postoperative	P
Lysholm scores	60,7±12.5	90,3±4.8	<0.05
Tegner activity scores	3,52±1,4	6.2±1.5	<0.05
Cincinati scores	14.8±5.3	26.9±1.6	<0.05

**Table 2:** Comparison of outcomes between work-accident and sports injury patients

Variables	Work-accident	Sports injury	P
N	16	17	
Preoperative Lysholm scores	59.1±11.2 (34-77)	62.1±13.8 (42-84)	0,417
Postoperative Lysholm scores	87.5±5.2 (78-95)	92.8±2.6 (90-100)	0,003
Preoperative Tegner activity scores	3.4±1.5 (1-6)	3.5±1.4 (2-7)	0,768
Postoperative Tegner activity scores	5.3±1.2 (3-7)	7.0±1.4 (5-9)	0,003
Preoperative Cincinati scores	14.0±4.7 (8-24)	15.7±5.9 (6-26)	0,376
Postoperative Cincinati scores	26.0±1.5 (24-29)	27.7±1.2 (26-30)	0,003
ROM deficits	-8.8±4.7 (-15-0)	-6.7±3.3 (-10-0)	0,001
Average time for returning to work	17.6±4.4 (12-27)	13.0±1.5 (10-15)	0,001

*N*=Number of patients, Postoperative and preoperative knee scores±standart deviation (Min-Max), ROM = Range of motion

**Table 3:** Comparison of outcomes between patients with or without meniscus lesions

Variables	With meniscus lesion	No meniscus lesion	P
N	25	8	
Preoperative Lysholm scores	59.9±11.4 (34-80)	63.0±16.0 (43-84)	NS
Postoperative Lysholm scores	90.3±4.9 (78-100)	90.2±4.9 (80-95)	NS
Preoperative Tegner activity scores	3.4±1.4 (1-6)	3.6±1.5 (2-7)	NS
Postoperative Tegner activity scores	5.8±1.4(3-9)	7.3±1.5 (5-9)	NS
Preoperative Cincinati scores	14.5±5.3 (6-26)	16.0±5.5 (8-24)	NS
Postoperative Cincinati scores	26.9±1.7 (24-30)	27.0±0.9 (26-28)	NS
Average time for returning to work	15.3±4.3 (10-27)	15.2±2.9 (12-21)	NS

Postoperative and preoperative knee scores±SD (Min-Max). *N*=Number of patients, NS = Nonsignificant (*P*>0.05), SD = Standard deviation

## Discussion

ACL injuries may be managed both conservatively and surgically. In patients who sustain a sedentary life, activity modification, muscle strengthening exercises, using braces and avoiding activities which need performance are preferred in the conservative management of ACL injuries [9, 10]. In these patients, the risk of developing osteoarthritis is increased by 50% in the next 10-20 years. Pain and loss of function occurs as a result of osteoarthritis [4]. However, ACL management has progressed toward surgical treatment in the last 10 years. The reasons for this are that the patients are young and active and they want to gain back their pre-injury activity levels and knee functions in the early period [11]. The patients included in this study were working underground, in the coal mines, in harsh conditions and usually on slippery surfaces. Additionally, because of the high collapse risk and mine-transportation vehicles, they were exposed to direct trauma. For patients, returning to active work early was important in the aspect of financial losses and continuation of their business contracts. Hence, in patients with poor knee stability (Lachman, pivot-shift +), conservative therapy was not recommended.

The most commonly and up-to-date method for ACL repair is the medial portal anatomical ACL reconstruction by hamstring autograft [5]. The rapid return of patients to their pre-injury activities and endeavors has gained importance. For this reason, the postoperative rehabilitation protocols have improved significantly and compelling early rehabilitation has started to gain more acceptance [7, 8]. Rapid return to sports is suggested especially within the postoperative 6 months following ACL reconstruction [12, 13]. Since our patients were young, active and working in harsh conditions, we recommended surgical treatment to them, also. We rapidly initiated the compelling early postoperative rehabilitation. We did not allow our patients to perform active sportive exercises for two months. The most important reasons for delayed returning to work in patients who had undergone ACL reconstruction for work-related accidents are the severity of the trauma and psychologic reasons. In these patients, posttraumatic stress disorder is seen more frequently when compared to sports injuries. In performed studies, although many factors have been reported to contribute for delay in returning to sports in athletes, the most important one was the psychologic factor [14–17]. The second factor may be secondary gain. Since these are legally work-related accidents, these patients cannot be laid off according to their employment contracts; situations occur in which large amounts of compensation become necessary to be paid by the employer. Atrophy of the quadriceps muscle is observed in most of the patients who undergo ACL reconstruction. In the literature, the most common complications have been reported to be muscular atrophy and weakness [18, 19] and insufficiencies in the strength of hamstring and quadriceps muscles, motor coordination and proprioception may continue in patients until the end of the 1st postoperative year [20].

The main reason for quadriceps weakness is not donor site morbidity, but the deterioration of the receptor and neuromuscular activation systems [21, 22]. Mendias et al. in their study, determined that myostatin and TGF-β levels were elevated in patients having muscular atrophy. Future studies may be conducted on myostatin [23]. In the study conducted by De Jong et al., they determined significant strength reduction in quadriceps muscle after ACL reconstruction with hamstring tendon graft. This reduction was being gradually less within 6-12 months postoperatively; however, when compared to the preoperative period, the insufficiency still continued [24]. Muscular atrophy was observed in 75% of our patients, and this was the main cause for delay in returning to work. This patient group also required physical rehabilitation therapy.

Physical rehabilitation plays an important role for achieving successful clinical outcome, following anterior cruciate ligament (ACL) reconstruction. To return to pre-injury activity level and sports, modern treatment methods and rehabilitation protocols have been widely realized and easily accessible [25]. Although variations exist among physical rehabilitation programs, accelerated programs are preferred to promote early return to sports and active living, which involve early initiation of motion, early regaining the muscle strength and recovery of knee functions [7, 8, 26–28]. We, also, used an accelerated physical therapy protocol in our clinic, immediately after surgery in our patients. Closed-kinetic chain exercises for strengthening quadriceps and hamstring muscle groups and exercises for joint motion range were routinely performed by all patients. Patients in whom insufficiency was thought to be present in extensor muscle groups were directed to receive professional physical rehabilitation therapy. We suggest that, in all patients who have undergone ACL reconstruction, physical rehabilitation by a professional team will increase the success rate of the operation and will accelerate the return to active life and work. In this study, the lack of a professional physical rehabilitation team to follow the patients postoperatively was a significant deficit. It might have been the reason for delayed return of patients to active life and work. In patients who had developed effusion and hematoma, return to work was delayed. In these patients, severe muscular atrophy developed and required professional physical rehabilitation therapy. The examination findings of the knee stability were normal in patients; however, since the duration for improvement of functional scores was long, they were delayed to return to work.

The association of meniscal and chondral injuries with ACL tear is frequently met and it has been shown to influence the postoperative healing process. Early surgical treatment, particularly in the first 6 months, has been suggested [29–31]. In patients with delayed ACL reconstruction who also had meniscal and chondral lesions, these injuries have been shown to have negative impact on the results of surgical treatment [32]. In our study, we determined that meniscal and chondral lesions had not influenced the return to work in the negative direction. The reasons for this result may be performing surgery in the early period, performing meniscal repair, and the repair of bad chondral lesions by mosaicplasty in three patients. Additionally, in these patients, not returning to work for 2 ½ months may serve as a period to complete the healing process of injuries inside the knee.

Single-legged hop test is the best testing method to assess knee functions for returning to sports in patients who have undergone ACL reconstruction [33, 34]. During decision-making process for returning to work in our patients, we allowed the patients who performed knee stabilization tests and single-legged hop test 75% and over to return to work. With tearing of ACL, the positional sense of the knee or proprioception is reduced [35]. In our study, evaluation of proprioception was not made; special exercise program for proprioception was not made, either. The most important limitation of our study was being retrospective. Other limitations were the lack of K-T dynamometry test, which is a knee stability test, the lack of professional physical rehabilitation for every patient and the lack of assessment and exercise program for proprioception.

## Conclusion

The most important factors which influence returning to work in miners operated for ACL are hematoma-effusion inside the knee, muscular atrophy and the long duration of rehabilitation therapy process. Since patients work in harsh conditions, they do not feel themselves ready for working. Therefore, patients who undergo operations for work-accidents are unwilling to get back to work. Physical rehabilitation therapy and psychological support should be given in these patients, similar to athletes, in order to reach their pre-injury activity levels.
